# Tissue characteristics and anatomic distribution of cardiac metastases among patients with advanced systemic cancer assessed by cardiac magnetic resonance (CMR)

**DOI:** 10.1186/1532-429X-18-S1-P265

**Published:** 2016-01-27

**Authors:** Shawn Pun, Andrew Plodkowski, Dipti Gupta, Yuliya Lakhman, Darragh F Halpenny, Matthew J Matasar, Angel Chan, Jennifer Liu, Richard Steingart, Jonathan W Weinsaft

**Affiliations:** 1 grid.51462.340000000121719952Cardiology, Memorial Sloan Kettering Cancer Center, New York, NY USA; 2 grid.51462.340000000121719952Hematologic Oncology, Memorial Sloan Kettering Cancer Center, New York, NY USA; 3 grid.51462.340000000121719952Radiology, Memorial Sloan Kettering Cancer Center, New York, NY USA

## Background

Cardiac metastases (CMET) impact management and clinical outcomes of patients with systemic neoplasms. CMR is well validated for evaluation of cardiac masses and increasingly used to assess oncologic patients, among whom pattern, tissue characteristics, and optimal diagnostic strategies for CMET are not known.

## Methods

The population comprised consecutive adults (≥18 yo) with metastatic systemic neoplasms who underwent contrast-enhanced CMR between 1/2012 - 8/2015. Patients with primary cardiac neoplasms were excluded. CMR was performed using 1.5T (88%) and 3T (12%) clinical (GE) scanners. A standard contrast-enhanced CMR protocol was applied: Cine-CMR (SSFP) was used to assess cardiac structure and morphology. DE-CMR (IR-GRE, TI 250-350 msec, 0.2 mmol/kg gadolinium) was used for tissue characterization; long TI (600 msec) DE-CMR was employed to confirm tissue properties of visualized masses. CMET was defined using established criteria as a discrete, irregularly contoured mass with discrete borders independent of cardiac chambers, myocardium, or central catheters. CMET was further categorized based on enhancement pattern (absent, diffuse, heterogeneous enhancement with patchy hypoenhancement). Transthoracic echocardiography (echo), if performed clinically within 30 days of CMR, was used to test conventional imaging for CMET.

## Results

115 patients (57 ± 15 yo, 54% male) with metastatic extra-cardiac primary neoplasms were studied; 29% (n=33) had CMET on CMR. Sarcoma (21% [n=7]) and melanoma (12% [n=4]) were the two leading primary cancer etiologies; atypical primaries also occurred (n=3 pancreatic, n=1 gastrointestinal stromal, n=1 CNS). CMET location markedly varied (45% RV | 27% LV | 18% RA | 12% LA | 27% pericardial); 21% of cases involved multiple cardiac locations. 76% were due to hematogenous or lymphatic spread; 24% were due to direct invasion. DE-CMR demonstrated CMET enhancement in 83% of cases; enhancement pattern was variable (54% heterogeneous, 46% diffuse). CMET often occurred in absence of pericardial (27%) or pleural (48%) effusions. 67% of the population underwent echo within 30 (6.7 ± 8.0) days of CMR, including 61% (n=20) of patients with CMET by CMR. As shown (Table 1), echo provided limited diagnostic sensitivity for CMET, whether assessed on a per-patient (75%) or per-location (74%) basis, despite excellent specificity (≥98%). Echo performance varied based on CMET morphology and location; CMET detected by CMR but missed by echo were either intra-myocardial (n=2) or in locations sub-optimally evaluated via transthoracic ultrasound (n=2 posterior LA | n=1 RV outflow tract).

**Table 1:** Diagnostic Performance of Transthoracic Echo for CMET as Established by CMR

	Sensitivity	Specificity	Accuracy	Positive Predictive Value	Negative Predictive Value
Echo (per-patient)	75% (15/20)	98% (55/56)	92% (71/77)	94% (15/16)	91% (56/61)
Echo (location-specific)	74% (17/23)	99% (354/357)	98% (371/380)	85% (17/20)	98% (354/360)

## Conclusions

CMET vary in location and enhancement pattern on CMR, often presenting without typical adjunctive findings such as pericardial or pleural effusions. Conventional screening via echo can be limited for CMET detection; incremental utility of CMR is typically provided for neoplasms that are intramyocardial or atypical in location.

